# From Memory to Attitude: The Neurocognitive Process beyond Euthanasia Acceptance

**DOI:** 10.1371/journal.pone.0153910

**Published:** 2016-04-18

**Authors:** Martin Enke, Patric Meyer, Herta Flor

**Affiliations:** Department of Cognitive and Clinical Neuroscience, Central Institute of Mental Health, Medical Faculty Mannheim, Heidelberg University, Mannheim, Germany; University of Udine, ITALY

## Abstract

Numerous questionnaire studies on attitudes towards euthanasia produced conflicting results, precluding any general conclusion. This might be due to the fact that human behavior can be influenced by automatically triggered attitudes, which represent ingrained associations in memory and cannot be assessed by standard questionnaires, but require indirect measures such as reaction times (RT) or electroencephalographic recording (EEG). Event related potentials (ERPs) of the EEG and RT during an affective priming task were assessed to investigate the impact of automatically triggered attitudes and were compared to results of an explicit questionnaire. Explicit attitudes were ambivalent. Reaction time data showed neither positive nor negative associations towards euthanasia. ERP analyses revealed an N400 priming effect with lower mean amplitudes when euthanasia was associated with negative words. The euthanasia-related modulation of the N400 component shows an integration of the euthanasia object in negatively valenced associative neural networks. The integration of all measures suggests a bottom-up process of attitude activation, where automatically triggered negative euthanasia-relevant associations can become more ambiguous with increasing time in order to regulate the bias arising from automatic processes. These data suggest that implicit measures may make an important contribution to the understanding of euthanasia-related attitudes.

## Introduction

There are many studies on peoples' positions on euthanasia. While several studies reported an increasing acceptance of euthanasia in Europe [1–3], the United States [4], and Asia [5–6], other studies investigating the same populations (Europe: [7–10]; United States or Asia: [11–12]) reported predominant opposition to the issue. Still others reported ambiguous results, in that attitudes are divided or undecided, or they vary over time [13–15].

Several reasons were discussed to explain these inconsistencies. Most importantly, euthanasia is not a specific term. It comprises different meanings and values, which are activated on different occasions and may conflict with each other [16]. Thus, different beliefs, values, and emotions influence the evaluation of euthanasia. To date, attitudes towards euthanasia were solely examined using explicit measures like questionnaires or scenarios. However, these measures are subject to conscious or unconscious bias in the participant, can be consciously influenced, and are affected by the diversity of activated beliefs, emotions and conations, especially in controversial issues [17]. Thus, more implicit methods are needed to understand the inconsistency in euthanasia research [16].

The use of implicit measurement techniques in attitude research aims to avoid response biases associated with explicit measures such as multi-item scales. In contrast to explicit measures of attitudes, which include self-report procedures, implicit measures are supposed to be inaccessible to self-report or introspection. They are characterized by not alerting the subject to the identity of the object of the attitude being measured [18]. The term "implicit" is prominent in recent discussions on indirect measures of attitudes and is also used to refer to implicit attitudes. However, this implies that individuals lack awareness of their implicit attitudes, which is misleading. What makes these measures implicit is that subjects may be unaware that their attitudes are being measured. That does not mean they are not aware that they possess those attitudes [19]. Hence, the terminology in this article is as follows: the term implicit measure is used to describe indirect measures. Implicit measures of attitudes provide estimates of an individuals' evaluation without them being aware they are giving information about their attitudes. These estimates are denominated an implicit attitude. The attitude object is the topic that is being evaluated and which is viewed either favorably or unfavorably. Implicit measurements require implicit tasks in which subjects are not aware that the function of the task is to measure attitudes (see [19] for an extended review of implicit measures).

Implicit measures of attitudes reflect automatic processes and are supposed to represent the cognitive laboratory equivalents of attitudes in real-life behavior, even when subjects are ambivalent or undetermined towards an attitude object [20]. Automatic processes are activated when subjects are confronted with an issue and automatically elicit positive or negative associations, which can be measured [21] and which are consistent estimators of the corresponding attitude [19, 22–23]. Furthermore, they can diverge from explicit measures, particularly when dealing with controversial attitude objects [24]. Hence, the comparison of implicit measures of euthanasia-related associations (implicit attitudes) and explicit attitudes is useful.

Attitudes towards euthanasia represent ingrained associations to positively or negatively valenced concepts in memory, which depend on prior learning experiences. Concepts and attributes that are continuously perceived in conjunction with an attitude object in everyday experiences are consequently associated with this attitude object by building neural interconnections in semantic networks [25–27]. Thus, the activation of the representation of the euthanasia object is supposed to activate associated concepts and attributes within this (neural) network (see [28] for spreading effects within memory), which are in the first instance independent of explicit memory processes [29] and therefore independent of diverging considerations, beliefs and ambivalences.

Behavioral measures only reflect the end product of cognitive processes [30]. Therefore, it might be advantageous to examine the cognitive process itself. A well-established approach is the evaluative (affective) priming paradigm and the use of event-related potentials (ERPs), since they reflect a temporally precise stream of electrophysiological activity that can be used to reveal the underlying cognitive mechanisms [31]. Two components have been predominantly explored in previous evaluative priming paradigms: the N400 and the late positive potential (LPP) [31–32].

The affective (evaluative) priming paradigm [21, 33] is a frequently used method in the investigation of attitudes. It examines whether the short presentation of a first stimulus, a prime, affects the processing of a subsequent stimulus, a target. The response-time to a valenced target is shorter after the previous presentation of an affectively related prime compared to an affectively unrelated prime. With regard to the evaluation of attitudes, the prime represents the attitude object and this facilitation effect of the prime to either positively or negatively valenced targets provides information about the preference for (or aversion to) the attitude object as an index for the real-life attitude [20, 34].

Two major theories are discussed for the cognitive process underlying affective priming. One account focuses on spreading activation processes similar to those occurring in semantic priming. The presentation of an attitude object (as prime) is assumed to automatically activate strong associations to that object like a spreading activation along the paths of memory-related networks, which include evaluative associations. In consequence, the activation levels of affectively related evaluations to an object are temporarily increased [21, 35]. A second account focuses on synergy or conflict processes in response tendencies during the evaluation of both prime and target. The prime automatically activates an evaluative response, which is either congruent or incongruent to the instructed response to the target. This response conflict model implicates that two independent responses are activated, one of which has to be inhibited and one has to be executed according to the instruction [20, 35]. Both theories refer to plausible mechanisms based on research (see [36] for extended comments).

The N400 is a negative deflection that generally peaks around 400 ms after stimulus onset. The N400 amplitude to a particular word was found to be highly sensitive to the immediate context in which these circumstances occurred. It has been shown to be reduced when the target word is semantically related to the immediately preceding prime relative to unrelated word pairs [37–38]. Thus, the modulation of this component reflects a facilitation effect in semantic retrieval. Its amplitude is larger over the right hemisphere for written words [30, 37]. A modulation of the N400 amplitude over frontal electrodes has also been found in evaluative (or affective) priming [35, 39–40] and has been supposed to reflect the facilitated cognitive effort when the evaluation of the target valence is congruent with the valence of the preceding prime. Because the modulation of the N400 is thought to reflect the influence of memory retrieval on early processes, the N400 is an important neurophysiological marker in examining attitudes and evaluative preferences.

The LPP is a positive wave with postponed peaking in affective processing [41] at 600 ms after stimulus onset (see [31]). Its amplitude is largest over centro-parietal electrode sites [42]. A number of studies demonstrated that the LPP is sensitive to the valence of a stimulus as found in affective priming paradigms. The LPP amplitude is increased when the valence of a target is affectively incongruent to the valence of an immediately preceding prime relative to congruent prime-target pairings [31, 39, 43–45]. Thus the modulation of the LPP reflects the cognitive effort in the affective evaluation of stimuli, which depends on the amount of incongruence to a preceding context. This might be due to increased attentional resource allocation during the evaluation process [39]. The LPP is thus well suited for the examination of attitudes, as it reflects the evaluative congruency of an attitude object relative to an affective context.

Whether the N400 or the LPP is more associated with affective priming is still an open question. Some studies found the modulation of the N400 amplitude to be sensitive to affectively incongruent prime-target pairs (lower amplitude) relative to congruent ones [40, 46]. On the other hand, some studies demonstrated that the modulation of the LPP is sensitive to evaluative incongruity in affective priming [44, 47] in that the LPP amplitude was shown to be more positive in affectively incongruent prime-target pairs. Thus, studies which investigated both the N400 and LPP as electrophysiological markers for affective incongruity are of special interest. The investigation of Zhang et al. [39] obtained a modulation of the N400 as well as of the LPP when pictures were used as primes and words were used as targets in an affective priming paradigm. However, Morris et al. [48] as well as Aguado et al. [32] obtained the modulation of the N400 in response to evaluative incongruity but not the modulation of the LPP. Herring et al. [31] reported the opposite results. In their three experiments they found a modulation of the LPP while the N400 was not influenced. Up to now, it remains unclear what drives the obvious differences.

In this study, the current experiment was conducted to understand the reasons for the ambiguous research outcomes in euthanasia acceptance. Furthermore, we aimed to provide an innovative and partly explorative approach to shed some light on the people’s predominant associations to euthanasia. Therefore, we examined the neurocognitive processes related to attitudes towards euthanasia with a focus on automatic associations and processes. These processes are captured by reaction time measures and electrophysiological data. In an explorative approach we examined both the N400 and LPP to determine the incongruity effect while euthanasia-related associations were activated during an evaluative priming paradigm. This allows us to compare implicit measures to self-report measures of attitudes towards euthanasia. We hypothesize that this is a crucial comparison as individuals use both explicit (controlled) and automatically triggered (unintended) processes to judge their environment [49] which supposedly diverge when different beliefs and emotions are linked to a controversial attitude object. Concordant with studies showing a difference between implicit and explicit attitudes (e.g. [23–24, 50]), we hypothesize that implicit associations towards euthanasia diverge from explicit considerations. Furthermore, the explicit measure is supposed to be ambiguous due to controversial considerations. The implicit measures should reflect associations towards euthanasia with identifiable positive or negative valence.

## Method

### Participants

Twenty right-handed medical students (ten female) ranging from 20 to 32 years of age (M = 24.1, SD = 2.8) were recruited from the two Medical Faculties of Heidelberg University, Germany. Another five participants were examined but had to be excluded (see below). All participants were native German speakers. Their vision was normal or corrected-to-normal. The participants were screened for a history of neurological and mental disorders, head surgery or injury. The study was approved by the Ethics Committee of the Medical Faculty Mannheim of Heidelberg University. All participants provided their written informed consent to participate in the study.

### Experimental tasks

**Fig 1:**
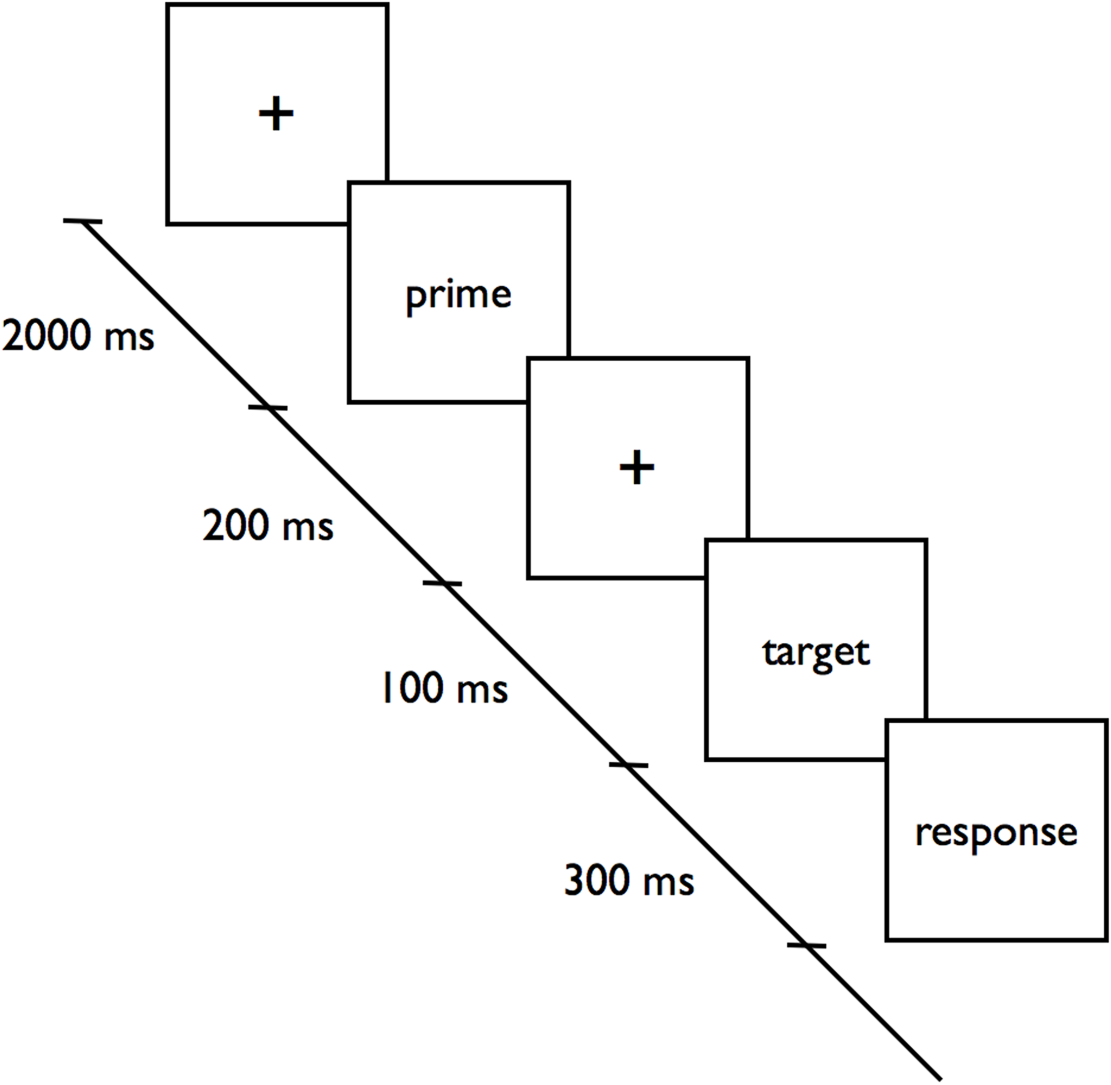
Time course of the affective priming paradigm.

All participants were confronted with an affective priming paradigm and were instructed to complete a questionnaire with multi-item scales. The time course of one trial of the affective priming paradigm is illustrated in Fig 1. One of three words appeared as a prime for 200 ms. A second word (target) was presented for 300 ms with a stimulus-onset asynchrony (SOA) of 300 ms. Participants were instructed to judge whether the second word (target) affectively fit or did not fit the first word (prime), using two response keys. The participants were also instructed to give their responses as quickly and accurately as possible. Most importantly, a cover story was used so that the subjects were not aware that this paradigm was being used to measure attitudes. They solved the task assuming that affective neural networks in memory were being investigated using an ambivalent medical stimulus (the word euthanasia).

All participants completed a questionnaire (modified from [51]) that measured their explicit attitudes towards euthanasia. Attitudes were examined using a 5-point scale ranging from 1 (totally disagree) through 3 (undecided) to 5 (totally agree). For each subject, the mean rating (Mx) was computed and transformed into a qualitative variable with the following domains: negative attitude (0 < Mx < 2.6), ambivalent (2.6 < Mx ≤ 3.4), positive attitude (3.4 < Mx < 5).

### Stimulus material

The affective priming paradigm consisted of 154 word pairs (all nouns; Table 1). The first word (prime) was either “sympathy” [German: “Sympathie”] (positive), “rejection” [German: “Abneigung”] (negative) or “euthanasia” [German: “Sterbehilfe”] (attitude object). Only these three primes were used due to the lack of adequate German synonyms for euthanasia and to avoid disproportions and perceptual interferences. The second word (target) was one of 77 negative nouns or 77 positive nouns, respectively. This resulted in 50 congruent (25 positive-positive & 25 negative-negative) and 50 incongruent word pairs (25 positive-negative & 25 negative-positive), and 54 word pairs related to the attitude object (27 euthanasia-negative & 27 euthanasia-positive). To avoid repetition effects, the target stimuli were presented only once. To reduce perceptual habituation to the three prime-words, every word pair was presented in random color and font. All 154 word pairs were counterbalanced across subjects. In order to create a list of 77 positive and 77 negative nouns, the Berlin Affective Word List Reloaded (BAWL-R; [52]) was used. We selected 154 nouns that did not have a semantic relation to euthanasia, illness or death. To avoid confounding factors in word processing, we controlled for arousal, vividness, word length, and word frequency on both lists.

**Table 1:** Stimuli and conditions of the affective priming paradigm.

Primes	Targets	condition	trials
“rejection”		25 negative-negative	
[“Abneigung”]	77 negative nouns	25 positive-positive	
“sympathy”		25 positive-negative	154 trials
[“Sympathie”]		25 negative-positive	
“euthanasia”	77 positive nouns	27 euthanasia-negative	
[“Sterbehilfe”]		27 euthanasia-positive	

### Procedure

After providing written informed consent, the participants were prepared for their EEG recording. They were seated in a comfortable chair 0.7 m in front of a 15-inch monitor in an insulated laboratory. The subjects were then instructed to solve the previously described priming task, using a “fit” and a “no fit” response key. Two orders were created to assign both keys to either the left or right side and counterbalanced across subjects. We chose this fit/no fit-instruction to enhance the relevant stimulus-response compatibility. This is an approach used to avoid the risk that participants ignore the repeatedly presented prime and it also enhances validity and reliability, however, the task might be less implicit (see [20]). Analyses were focused on the comparison of the four conditions evolved from the stimulus material: 1) ‘congruent’ (positive-positive and negative-negative prime-target pairs averaged), 2) ‘incongruent’ (negative-positive and positive-negative prime-target pairs averaged), 3) ‘euthanasia-negative’ (pairings of the word euthanasia as prime and negative targets), and 4) ‘euthanasia-positive’ (pairings of the word euthanasia as prime and positive targets). Reaction times and type of response were chosen for analysis. Only trials with "fit"-responses were included in the congruent condition and "no fit"-responses in the incongruent condition. In the euthanasia-conditions it was not possible to determine congruence without the participants' response, because subjects could rate euthanasia as either positive or negative according to their attitude. Thus, the frequency of the two different responses was analyzed. The response with the higher frequency was determined as correct. Subjects were then grouped according to their response proportions: more "fit"-responses in the euthanasia-positive condition (n = 5) or in the euthanasia-negative condition (n = 20). Analyses were limited to the sub-sample of 20 subjects to ensure that the same cognitive process was examined (i.e., subjects had a consistent and not undecided response sequence). Unfortunately, a comparison of both groups was not feasible due to the small group size of one sub-sample. The completion of the questionnaire occurred only at the end of the testing after the cover story has been uncovered to ensure that the indirect measures were not affected by the explicit interrogation of attitudes in the questionnaire.

### Electroencephalographic recordings and processing

Electroencephalographic data were recorded from 60 equidistant scalp sites using sintered Ag/AgCl-electrodes. All electrodes were referenced online to the left mastoid. AFz served as ground. Electrooculographic activity (EOG; vertical (VEOG) and horizontal (HEOG)) was measured. Impedances were maintained below 5 kΩ. BrainAMP amplifiers (Brain Products, Inc., Munich, Germany) were used to amplify EEG and EOG data with a sampling rate of 250 Hz. Brain Vision Analyzer Software Version 1.05 (Brain Products, Inc., Munich, Germany) was used for data reduction. The raw data were referenced to the mathematically linked mastoids [53] and filtered with a band pass of 0.1 to 30 Hz (24 dB/octave). An Independent Component Analysis (ICA) was applied to separate and remove unstable confounding factors arising from eye blinks and other stereotypic artifacts (e.g., horizontal eye movement, heart beat artifacts, muscle artifacts) by linear decomposition [54–55]. Epochs were segmented as follows: the critical interval started 100 ms before the prime and ended 1600 ms after the prime. Similar to the reaction time analyses, only epochs containing a “fit”-response in the condition euthanasia-negative and a “no-fit”-response in the condition euthanasia-positive were selected for the ERPs analysis. This response-logged analysis explains the maybe unusual long epoch. These Segments were baseline corrected to the mean of the 100 ms pre-stimulus period. Separate ERPs were constructed by averaging the corresponding trials to each condition and response.

Since the EEG analysis should primarily capture the deflections of the N400 and the LPP component, the time windows were trimmed post-hoc by visual inspection of the grand average ERPs. The N400 was quantified by computing the mean amplitude from 380 to 420 ms after target onset. The mean amplitude of the LPP ranged from 650 to 950 ms after target onset. N400 analyses were limited to the averaged array of the five right fronto-lateral electrodes (AF4, F2, F4, FC2, and FC4) as previous research suggested a right-frontal distribution when written words and valenced stimuli are used [30–31, 37, 56–57]. The LPP analyses were limited to the averaged array of the six parietal electrodes (P1, Pz, P2, PO3, POz, and PO4) consistent with the previous literature [42, 58–59].

### Statistical analysis

A one-way factorial repeated-measures analysis of variance (rmANOVA) was performed with the factor ‘condition’. This factor comprised four levels equivalent to the four different conditions of prime-target pairings (euthanasia-positive, euthanasia-negative, congruent, incongruent), which were presented to the participants. This one-way factorial design was conducted to compare the N400, LPP, and RT measures. In addition to the comparison of the critical conditions euthanasia-positive and euthanasia-negative with each other, we contrasted them with the baseline conditions congruent and incongruent. In case of a statistically significant within-subjects effect, we performed post-hoc analyses to identify the source of the main effect. We used a significance level of p< .05. Effect sizes were reported using partial eta square (η^2^). If the assumption of sphericity was violated, F-values were Greenhouse-Geisser corrected according to epsilon (ε).

## Results

### Questionnaire data

Most subjects (65%, n = 13) showed an ambivalent rating, 30% (n = 6) rated euthanasia as positive and only one subject (5%) as negative. On average, euthanasia was evaluated as ambivalent with a slight positive tendency (M = 3.38, SD = 0.43, range 2.8–4.6).

### Reaction time data

**Fig 2:**
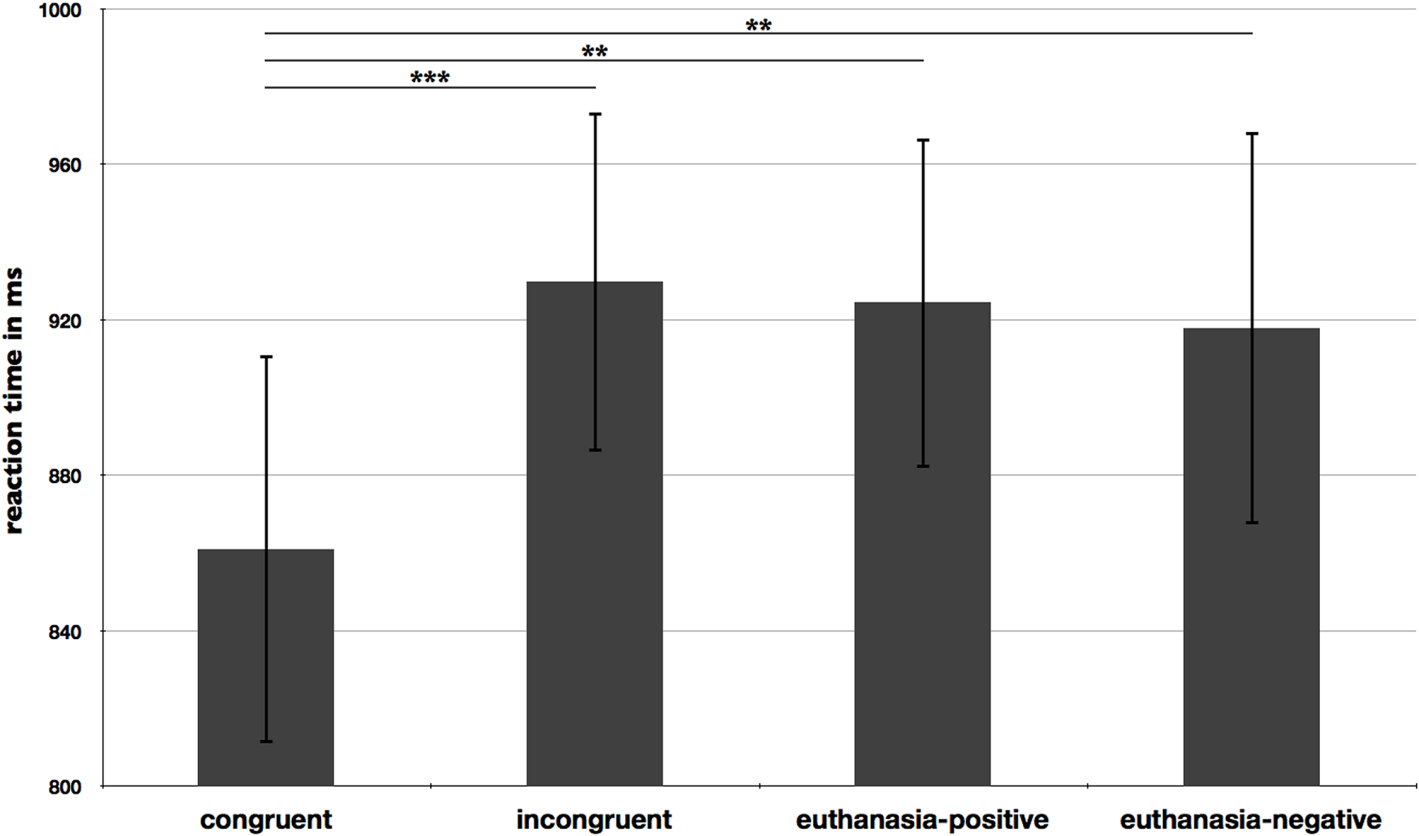
Mean reaction times in milliseconds (ms) for the conditions congruent, incongruent, euthanasia-positive, and euthanasia-negative. Error bars reflect one standard error. Note: *** p < .001, ** p < .01, * p < .05.

The repeated-measures ANOVA revealed a significant main effect of condition (F(1.55,29.45) = 4.54, p = .027, ε = 0.517, partial η^2^ = .193). Post-hoc analyses showed that the mean reaction times of the conditions ‘congruent’ and ‘incongruent’ differed significantly (Fig 2, t(19) = -4.47, p ≤ 0.0001). Participants showed faster reactions in congruent prime-target pairs (M = 861.02, SD = 223.71) compared to incongruent pairs (M = 929.86, SD = 195.58). However, the mean reaction times of the conditions ‘euthanasia-positive’ and ‘euthanasia-negative’ did not differ significantly (p = .834). Differences as well as between ‘incongruent’ vs. ‘euthanasia-positive’ and ‘incongruent’ vs. euthanasia-negative also failed to reach statistical significance (p > .05), in contrast to ‘congruent’ vs. ‘euthanasia-positive’ (t(19) = 2.59, p = .018) and ‘congruent’ vs. ‘euthanasia-negative’ (t(19) = 3.68, p = .002).

### ERP results

#### N400

**Fig 3:**
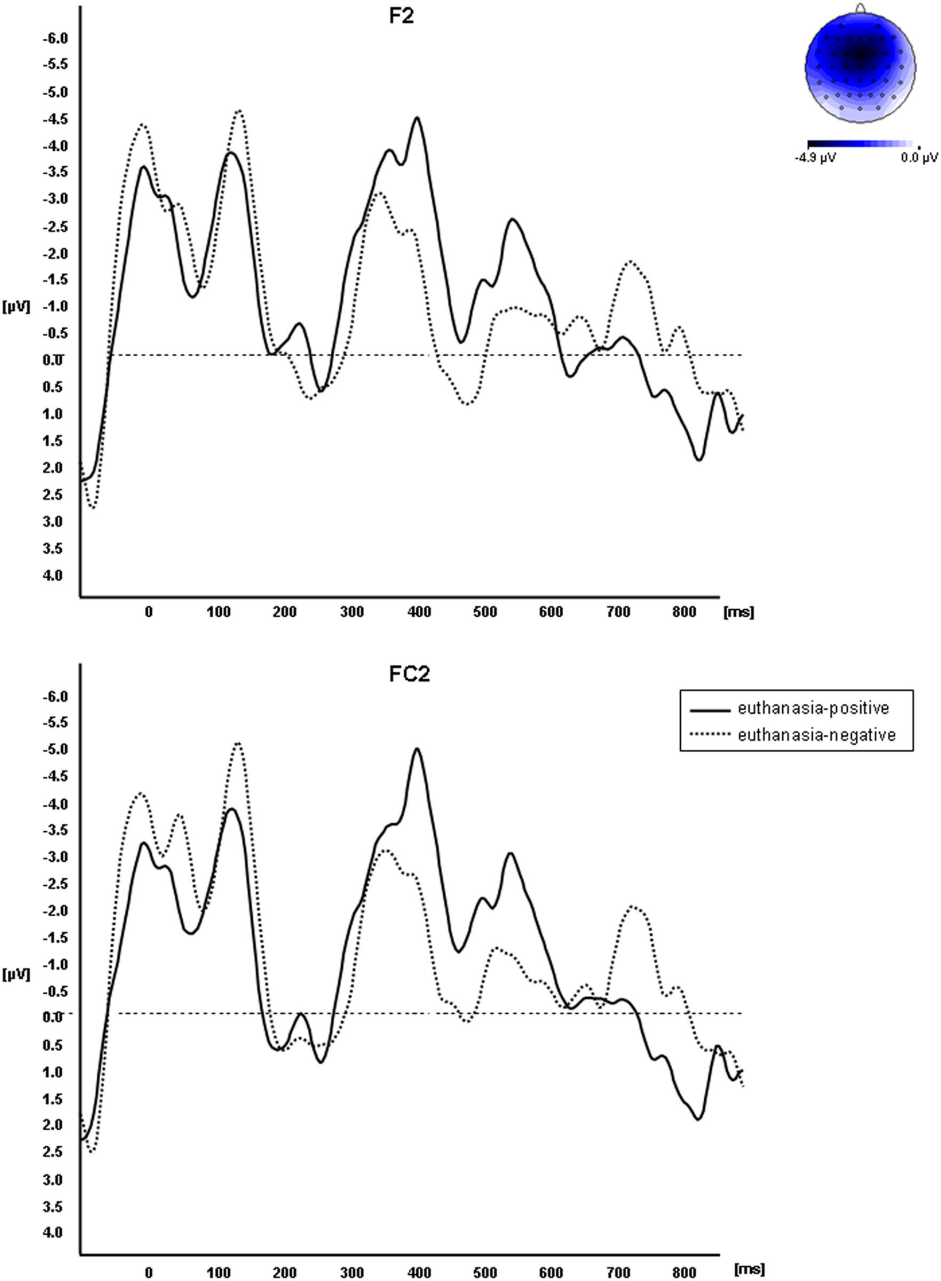
Effects of response facilitation upon the N400 amplitude at two exemplary right fronto-lateral electrode sites (F2 and FC2) around 400 ms after target onset. The amplitude of the dashed waveform, reflecting the response to negative targets, is significantly lower than the solid waveform, reflecting the response to positive targets, when the word euthanasia acted as prime. In the upper right the scalp distribution of the difference between euthanasia-positive and euthanasia-negative between 380 to 420 ms is mapped.

The repeated-measures ANOVA revealed a significant main effect of condition (F(3,57) = 4.36, p = .008, partial η^2^ = .187). Post-hoc analyses showed that the (negative) ERP mean amplitude in the condition ‘euthanasia-negative’ was significantly lower than in the condition ‘euthanasia-positive’ (t(19) = -2.59, p = .018; Fig 3). The mean amplitudes in the conditions ‘congruent’ vs. ‘incongruent’ did not differ significantly (t(19) = .17, p = .868). While the mean amplitude in the condition ‘congruent’ was significantly lower than that in ‘euthanasia-positive’ (t(19) = -2.31, p = .032), there was no significant difference between ‘congruent’ vs. ‘euthanasia-negative’ (t(19) = .66, p = .515). However, the mean amplitude in the condition ‘incongruent’ was also significantly lower in comparison to ‘euthanasia-positive’ (t(19) = -2.65, p = .016) and was also not significantly different from ‘euthanasia-negative’ (t(19) = .75, p = .462).

#### LPP

The repeated-measures ANOVA did not reach statistical significance in the comparison of all levels of the factor condition (F(1.58,30.04) = 1.49, p = .240, ε = .527, partial η^2^ = .073).

## Discussion

In this study we examined explicit attitudes towards euthanasia as well as reaction time measures and both the N400 and LPP component during an evaluative priming paradigm. The latter measures served to determine the relationship of automatically triggered euthanasia-related affective associations and explicit attitudes towards euthanasia.

In their explicit evaluation of euthanasia, medical students were predominantly undecided in their acceptance of or opposition to euthanasia. The mean rating of all participants was ambivalent but had a slight tendency towards acceptance. This is in line with the inconsistent results in systematic reviews and meta-analyses [60–61]. On the behavioral level, reaction times in the priming paradigm were significantly shorter for congruent prime-target pairings than for incongruent pairings. This replicates previous reports on affective priming (see [62–63] for reviews). However, reactions to positive and negative words were not differentially influenced by euthanasia as prime. This might be an indication of ambivalence. On the electrophysiological level, no significant modulation of the LPP component was found. In contrast to previous reports of a modulated LPP amplitude in affective incongruity [31, 39, 44–45], the LPP amplitude did not differ significantly in response to positive or negative targets, neither when a positive or negative word nor when the word euthanasia was used as prime. The electrophysiological results revealed effects on the N400 component at right fronto-lateral scalp locations between 380 and 420 ms after target onset. The significantly lower N400 amplitude in the condition ‘euthanasia-negative’ in contrast to ‘euthanasia-positive’ indicates a facilitated integration of negative words when they are immediately preceded by the word euthanasia. Concordant with previous findings of reduced N400 amplitudes (e.g. [35, 39, 40, 48]) this reduction reflects decreased cognitive effort in evaluative processes when the target valence is congruent with the formerly primed context. This suggests that the word euthanasia was more ingrained in negatively valenced networks in memory compared to positively valenced ones.

Although we limited our analyses to the sub-sample of subjects who consistently classified the word euthanasia as negative in the priming task, the questionnaire ratings tended to yield more positive statements. The explicit evaluation of the euthanasia issue might deviate from automatically triggered associations as a result of multiple considerations and reflections of social norms and prevailing opinions in society. This supports the idea that the explicit evaluation only reflects a compromise of appreciated considerations within a dynamic process of thinking about euthanasia, which in turn handicaps the formation of a clear position (see also [13–15]).

Whereas semantic priming is a robust phenomenon, affective priming is not always observed (cf., [62–63] versus [64–65]). Stimulus detection is modulated by arousal and valence. The allocation of cognitive resources to highly emotional stimuli is sped up, but in turn, participants also need time to shift their attention away from highly emotional words [66]. It is not clear whether the extreme word euthanasia is so acutely emotional and arousing that it binds attention or if it elicits a more conscious processing and therefore biases the reaction to the following target equally for both positive and negative words. In some studies, reduced priming effects were obtained when extremely negatively valenced and arousing primes were used [67–69]. This finding has been labeled “contrast effect”. Such effects could also have been elicited in trials with an extreme prime [70]. As diminished priming effects have been attributed to increased corrective efforts (e.g. [71]), contrast effects are likely to occur when automatic evaluations become conscious. Given the large range of reaction times on the behavioral level, inferences with conscious and corrective processes like controversial considerations about social norms and prevailing opinions are possible. This could explain the lack of an affective priming effect in the euthanasia conditions as the RT measures reflect more explicit processes.

The number of trials used in an affective priming paradigm is an important factor for success in obtaining a priming effect. Too few trials are likely to produce unstable effects, whereas a large number of trials are likely to diminish the impact of the prime [19]. This might be a reason why we failed to demonstrate a behavioral effect in the euthanasia conditions, but obtained an effect in the congruent condition, which comprised nearly twice as many trials. Similarly, in the ERP data, the amount of added EEG segments aggregated to compute the grand average was smaller in the euthanasia conditions than in the other conditions. The lack of behavioral and LPP priming effects in the euthanasia condition could thus be related to a lack of power.

Significant ERP priming effects in the absence of behavioral priming effects have also been reported in previous studies (e.g. [32, 47, 72]). This might be due to the different time frames and cognitive sub-processes they reflect.

Before we refer to the ERP effects we have to critically address several limitations. Due to missing detailed a priori assumptions with regard to the selection of exact time windows used in the ERP statistics, our electroencephalographic analyses were partly of exploratory nature. We used post-hoc analyses and visual inspection of recordings to construe the time windows of interest. This selection of time windows needs to be validated in future experiments. The exploratory character has to be considered in the discussion and conclusions.

In contrast to previous reports of a modulated LPP component in affective incongruity [31–32, 39, 44–45], we failed to demonstrate such an effect. This might be due to the applied word-word paradigm, which is considerably different to previous research. Previous LPP findings in affective priming are predominantly based on picture-word paradigms. Other studies also failed to obtain an LPP effect using a word-word paradigm (e.g. [40]). An LPP effect in affective priming might depend on the stimuli used in the paradigm. Zhang et al. [39] argue that their obtained LPP effect relies on picture processing during their picture-word paradigm. This might explain the lack of an LPP effect in our study. Since the LPP component also reflects attention resource allocation [39, 41–42], the characteristics of stimuli in an affective priming paradigm have to be addressed in more detail in future experiments (cf. [31]).

As the LPP component also reflects cognitive processing resources and top-down regulatory processes, the LPP could indicate a more conscious processing of prime and target (see also [39]). Consistently, the LPP effect was not obtained when primes were subliminally presented in an affective picture priming paradigm [73]. The LPP component between 650 and 950 ms after target onset might thus reflect a stage of evaluation within the neurocognitive process, which is affected by conscious top-down regulations similar to those assumed to cause the similar reaction times (approx. 920 ms after target onset) in the euthanasia conditions. These conscious regulations or modulations of a formerly automatically triggered process may stem from the ambivalence we measured on an explicit questionnaire level comprising multiple considerations and reflections of social norms and the prevailing opinions in society. The fact that we found a priming effect (for "euthanasia" and negative words but not for "euthanasia" and positive words) in the early N400 component in contrast to the later LPP component and RT measures supports this idea. We hypothesize that the level of conscious control constitutes the significant factor which accounts for the difference between the LPP and N400 component. The N400 reflects an earlier cognitive process of associative memory involvement, which, supposedly, is not affected by ambivalence and conscious top-down regulation. Interestingly, Aguado et al. [32] also found a significant N400 effect for affective incongruity in the absence of significant behavioral priming and LPP effects. Other studies registered a significant LPP effect for affective incongruity [31, 39, 43–45], however, they all applied stimuli that are not affected by ambivalence and the need for conscious top-down regulation.

We suppose that the N400 indexes a more context-dependent process similar to its role in semantic priming. Accordingly, the modulation of the N400 reflects an integrative process by which the target is integrated into the preceding context to gain a unified representation of prime and target (see also [32]). The deflection of the N400 to incongruent or unexpected words is modulated by the predictability of items in the affective context and is assumed to reflect an enhanced effort to activate a word’s concept when no contextual/affective pre-activation in semantic memory has taken place. Thus, the prime pre-activates an affective network in memory and facilitates the responses to targets, which are affectively related to the primed context, which is then reflected by lower N400 amplitudes. The obtained deflection of the N400 in our experiment shows that euthanasia is clearly embedded in a negatively valenced memory network built-up through our years of experience with the world and can therefore be automatically afflicted by negative associations via spreading activation. However, recently it has been argued that a modulation of the N400 component can be also caused by familiarity-related episodic memory retrieval processes ([74]; see [75] for an opposite view). Although our design, which is not a masked priming design, or a lexical decision task is not specially constructed to differentiate between those two possibilities, we think that the reported difference between positive and negative target items cannot be explained by differences in familiarity alone as all items should be familiar to an identical degree.

A potential explanation of the diverging results could be the use of the compound word "euthanasia" itself. This term is ambiguous as its terminology implies "a good death" (see [16]). Also, the German word "Sterbehilfe" literally comprises the words "die" and "help", which are contrarily valenced. In consequence, it is difficult to ensure that it is equally implicitly processed by each subject. However, in semantic priming, an N400 effect was observed even when complex sentences were processed [37]. Whether the complexity of the compound word "euthanasia" affects implicit processing and therefore biases the deflection of the N400 in the affective priming paradigm has to be considered in future experiments.

A possible cause of the N400 effect we have to consider and a limitation of our design is the applied fit/no-fit instruction in the affective priming paradigm, which is more prevalent in semantic priming (e.g. [27]). De Houwer [20] argued that participants are likely to ignore the prime or target word, especially when the prime or target is capable of distracting them from the task. This is a considerable problem, since there is no synonym for the word 'euthanasia' in the German thesaurus and we were forced to repeatedly use this single word as prime. We applied the fit/no-fit instruction (with regard to the affective content of prime and target) to prevent participants from ignoring or habituating to the word euthanasia. This kind of instruction is assumed to enhance stimulus-response compatibility (stimulus features determine a related response), and, consequently, to enhance validity and reliability (see [20] for extended arguments). However, instructions with irrelevant stimulus-response compatibility were usually applied in affective priming paradigms [63]. It may be that the obtained N400 effect is due to match/mismatch processes rather than spreading memory activation (see also [27]), which requires additional research for clarification. Nevertheless, the prime and target words were free of semantic relation since we controlled for that.

Two major theories are discussed to explain the cognitive process underlying affective priming. One account focuses on spreading activation along the paths of memory-related networks, in which the prime is assumed to automatically activate strong evaluative associations (see [21]). A second account is the response conflict model. The prime is assumed to automatically activate a response to the prime, which is either congruent or incongruent to the response to the target (see [35]). Our design cannot contribute extensively to that discussion but our data favour the spreading activation model. According to response conflict research a stimulus set is presented which activates independent responses. One automatically activated response has to be inhibited and one central response has to be executed based on the task instruction. This only occurs in tasks with irrelevant stimulus-response compatibility [20], in which the prime is not part of the instruction. By contrast, in the present study, there was no irrelevant stimulus because both prime and target were part of the instruction and had to be compared. Thus it is unlikely that two independent responses were activated, which argues against the response conflict model (see also [27]). Given our design and the obtained priming effects, our data favour a spreading activation model of memory processes. However, additional research will be needed for clarification. At this time, we suggest that the word euthanasia activates memory-related networks that comprise strong evaluative associations, which originate from associative learning.

## Conclusion

**Fig 4:**
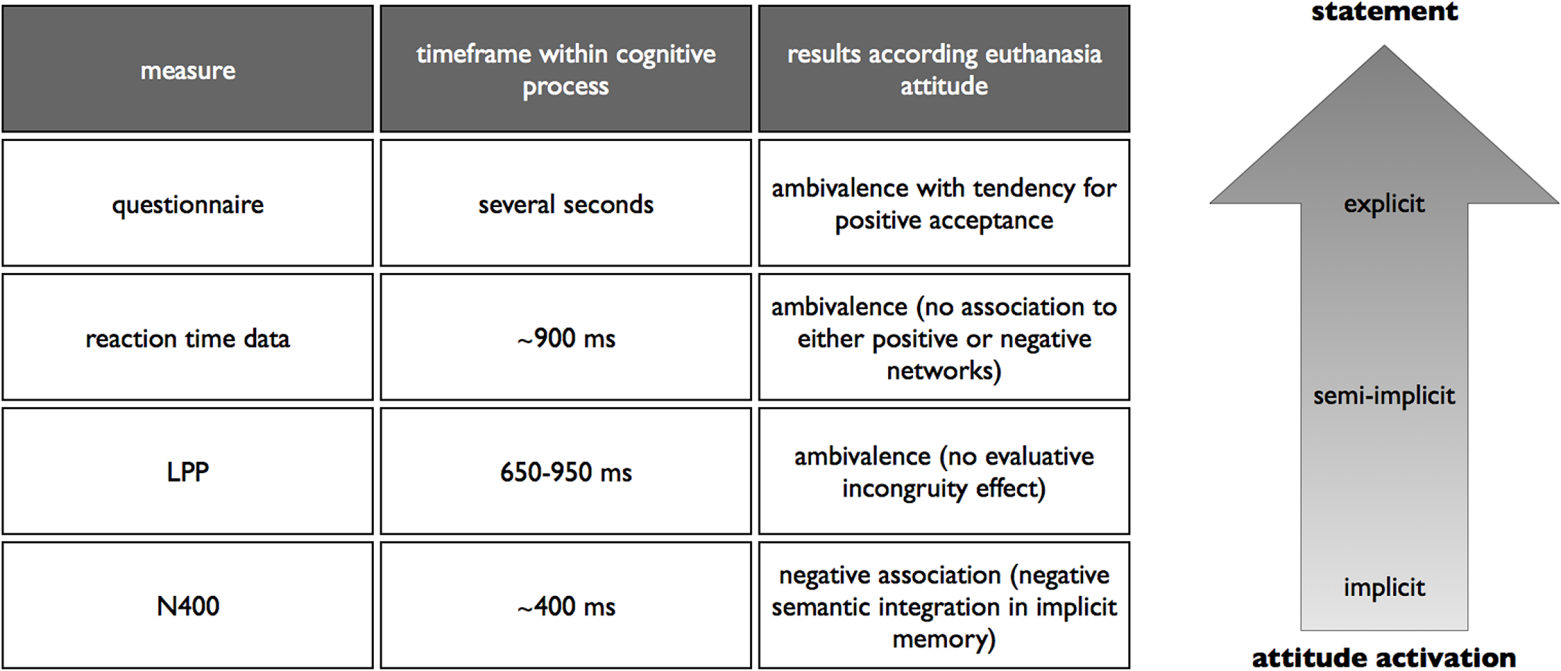
Integration of all measures.

The integration of all measures suggests a bottom-up process of attitude activation, where automatically triggered negative euthanasia-relevant associations in memory are activated and become more ambiguous with increasing time to control or correct this arising process due to top-down regulations (Fig 4). We assume that an explicit statement reflects the end product of evaluations, which are automatically activated and are based on ingrained associations in memory, which efficiently align individuals with their environment (to favor or oppose sth.) without need for conscious considerations (see [76]). However, we furthermore assume that automatic evaluations of euthanasia are controlled by the prevailing ethical discussions and social norms, as well as the terms of social desirability. Therefore bottom-up and top-down processes are both used to evaluate the complexity of the attitude object (see also [24, 49]) but might work in a constraining manner (see [37]). This results in ambivalent measures due to increased regulatory capacity over time. Further research in controversial attitude objects should consider implicit measures as an indispensable tool in addition to behavioral measures and the temporal placement of their measures within the dynamic neurocognitive process of attitude activation.
